# CAPRG: Sequence Assembling Pipeline for Next Generation Sequencing of Non-Model Organisms

**DOI:** 10.1371/journal.pone.0030370

**Published:** 2012-02-03

**Authors:** Arun Rawat, Mohamed O. Elasri, Kurt A. Gust, Glover George, Don Pham, Leona D. Scanlan, Chris Vulpe, Edward J. Perkins

**Affiliations:** 1 Center for Pathogen Information, Translational Genomics Research Institute North, Flagstaff, Arizona, United States of America; 2 Department of Biological Sciences, University of Southern Mississippi, Hattiesburg, Mississippi, United States of America; 3 Environmental Laboratory, EP-P, U.S. Army Engineer Research and Development Center, Vicksburg, Mississippi, United States of America; 4 School of Computing Science, University of Southern Mississippi, Hattiesburg, Mississippi, United States of America; 5 Department of Nutritional Science and Toxicology, University of California, Berkeley, California, United States of America; Auburn University, United States of America

## Abstract

Our goal is to introduce and describe the utility of a new pipeline “Contigs Assembly Pipeline using Reference Genome” (CAPRG), which has been developed to assemble “long sequence reads” for non-model organisms by leveraging a reference genome of a closely related phylogenetic relative. To facilitate this effort, we utilized two avian transcriptomic datasets generated using ROCHE/454 technology as test cases for CAPRG assembly. We compared the results of CAPRG assembly using a reference genome with the results of existing methods that utilize *de novo* strategies such as VELVET, PAVE, and MIRA by employing parameter space comparisons (intra-assembling comparison). CAPRG performed as well or better than the existing assembly methods based on various benchmarks for “gene-hunting.” Further, CAPRG completed the assemblies in a fraction of the time required by the existing assembly algorithms. Additional advantages of CAPRG included reduced contig inflation resulting in lower computational resources for annotation, and functional identification for contigs that may be categorized as “unknowns” by *de novo* methods. In addition to providing evaluation of CAPRG performance, we observed that the different assembly (inter-assembly) results could be integrated to enhance the putative gene coverage for any transcriptomics study.

## Introduction

With the advent of next generation sequencing (NGS) [1], application of transcriptomics to address biological questions in non-model organisms has grown phenomenally [2], [3]. Although the generation of sequence data for non-model organisms continues to accelerate, the development of assembled transcriptomes and genomes for these organisms remains challenging [2]. Most transcriptomics studies for non-model organisms use sequence assembly as a first step to generate contiguous sequences (contigs) which consist of overlapping reads that provide a consensus-based full length transcript. Multiple algorithms for *de novo* alignment have been developed including: overlap-layout-consensus (OLC) strategy which is used in CAP3 software [4] and PHRAP [5]. Alternatively, graph methods based on suffix trees [2] have been employed for alignment in NEWBLER (454 Life Sciences, Branford, CT) and VELVET [6] algorithms. The increasing number of sequence reads and longer read length created by the latest generation of sequencers will require high computational memory and management to achieve sensitivity, accuracy and timeliness of assembly. Due to n^2^ complexity of the OLC [7], [8], the memory requirement for the NGS reads are high relative to graph methods. To keep pace with increasing sequence read length, assembly with OLC can be performed with different tools/pipelines to manage memory such as TGICL [9] and PAVE [10], which use clustering with megablast followed by assembling. Alternate methods like MIRA use hybrid strategy for high and low confidences regions and take SNPs into account [11]. Graph based methods like VELVET and SOAP Denovo [12], [13] that rely on K-mer are considered to be less memory intensive [7]. VELVET and MIRA can perform assembling on both long reads as well as short reads while SOAP Denovo works on short reads.

Next generation sequencing technologies including Illumina (Illumina, Inc., San Diego, CA), SOLiD (Life Technologies Corporation, Carlsbad, CA) and Helicos (Helicos BioSciences Corporation, Cambridge, MA) generate millions to ten's of millions sequence reads (40–200 bp) per run representing immense data content [14]. As next generation sequencing technology continues to develop, the length of reads will likely increase [15] as has been observed with the 454/Roche platform which initially yielded 100 bp reads and now consistently yields >400 bp reads. The alignment of short sequencing reads generated by NGS to a reference genome has been successfully applied to reads less than 200 bp [15], [2]. Examples of these short read assembly algorithms include SOAP [12], [13], MAQ [16], BOWTIE [17], BWA [18], Novoalign [19], STAMPY [20] and ABYSS [21]. Additionally, BLAT [22], SSAHA2 [23] and BWA-SW [15] have been used to align long reads/contigs against a reference genome. By comparison, traditional long sequence read alignment programs are relatively slow when compared to short read aligners [15]. With the introduction of BWA-SW [15], the alignment of long reads can be done faster in comparison to other long read alignment programs such as BLAT and SSAHA2 [15].

**Figure 1:**
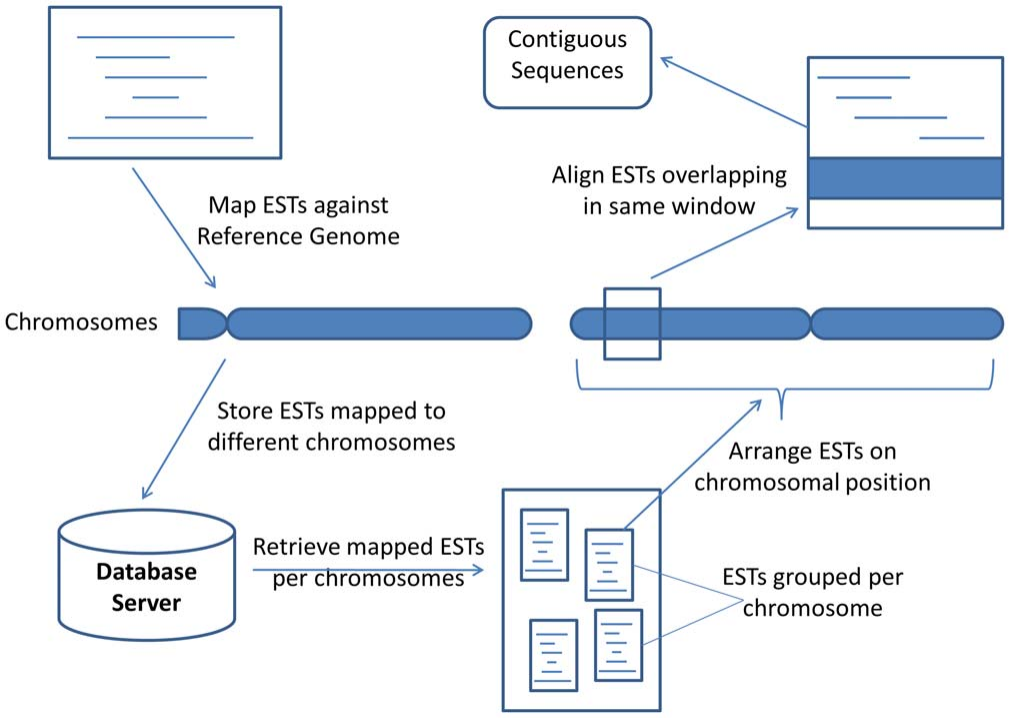
The flow chart of CAPRG representing the mapping of reads to generate contiguous sequences (contigs).

Mapping reads to a divergent reference genome represents an alternative to *de novo* assembly as the read length increases, especially for large genomes that have high repetitive sequences [20]. New approaches where a reference genome can be leveraged as scaffolding for assembling the novel genome of interest have been applied [24]. Based on a similar concept, we introduce the ‘Contig Assembly Pipeline against Reference Genome’ (CAPRG) which first maps “long reads” against the reference genome followed by assembly, instead of *de novo* assembly approaches followed by conventional pipelines and tools (Figure 1). In the first step of CAPRG alignment, ESTs juxtaposed to “one another” spanning “across” the chromosome are first sorted with an anchor position on the chromosome. Groups of ESTs are binned to a common window based on its anchor position to the chromosome. Within each window, these groups of ESTs are assembled and contigs are generated under criteria of high percentage of identity. This new approach was assessed by assembling the non-model organisms Japanese quail (*Coturnix japonica*) and Northern bobwhite (*Colinus virginianus*) against the closely-related phylogenetic relative, the chicken reference genome. Chicken (*Gallus gallus*) and Japanese quail belong to same family Phasianidae while Northern bobwhite is more distant to chicken and belongs to family Odontophoridae. Finally, to assess the effects of increased phylogenetic distance from the reference genome on sequence assembly using CAPRG, the zebra finch (*Taeniopygia guttata*) genome which belongs to distantly related taxonomic order Passeriformes was used as a reference to assemble the transcriptome of Northern bobwhite and Japanese quail.

## Results and Discussion

Most transcriptome projects for non-model organisms focus on maximizing the number of genes found, often termed as ‘gene hunting’, and minimizing the number of redundant contigs [3]. As described in the introduction, a number of de *novo* assembly methods including OLC and graph methods have been developed to achieve these ends. We have developed an alternative approach (CAPRG), which utilizes a reference genome to build sequence clusters for sequence assembly. The performance of the *de novo* assembly methods including OLC (PAVE), graph method (VELVET) and hybrid assembler (MIRA) were compared with our reference-based OLC assembly tool, CAPRG.

Assembler parameterization has been shown as an important step in determining the output of an EST project [3]. The output of a graph-based method such as Velvet is highly dependent on K-mer size while OLC assemblers such as CAP3 are affected by percent identity. We took into consideration the parameter space for the assembly output with different methods. We then compared the output of these different assemblers against CAPRG. The measurement of the assembly output can be done by the size and accuracy of their contigs [7]. We first established benchmarks for assembly output based on the number of contigs and average length instead of using N50 because N50 statistics for different assemblies are not comparable [7]. Secondly, we used the annotation based on homology and more stringent reciprocal blast hit (RBH) to evaluate the redundancy factor of the contigs generated by each assembly method relative to the total number of unique homologs. To assure an unbiased comparison of results among assembly methods, same input files for each species and equivalent BLAST cutoff values for matches were set among assembly runs.

**Figure 2:**
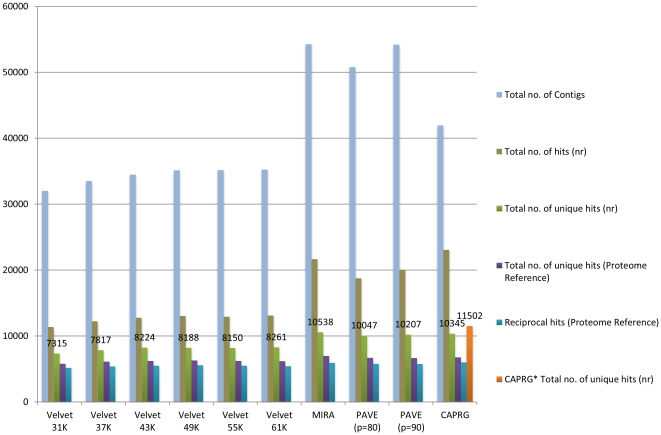
Assembly comparison for *Colinus virginianus* using various assembler programs and parameter spaces. *E-value* cutoff for all database searches was <10E-05. The abbreviation “nr” represents non-redundant protein database from NCBI and “K” represents K-mer size. CAPRG* are assembly of reads that mapped to reference genome singly or failed to assemble by windowing against chromosome.

**Figure 3:**
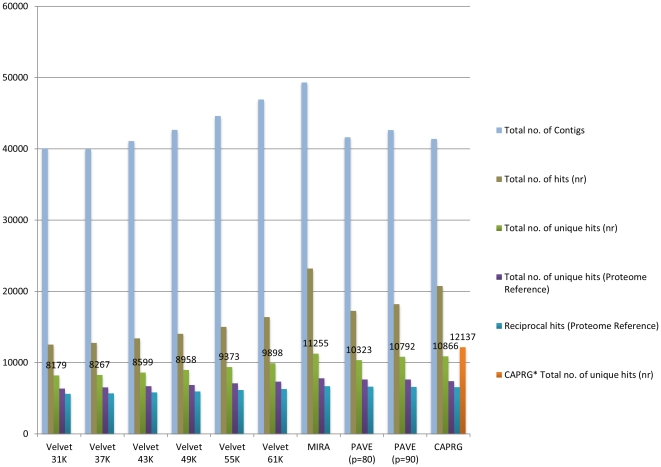
Assembly comparison for *Coturnix japonica* with various assembler programs and parameter spaces. *E-value* cutoff for all database searches was <10E-05. The abbreviation “nr” represents non-redundant protein database from NCBI and “K” represents K-mer size. CAPRG* are assembly of reads that mapped to reference genome singly or failed to assemble by windowing against chromosome.

Given that graph-based assembly algorithms such as VELVET use K-mer similarity to determine sequence homology, the computational cost of algorithm execution is significantly reduced due to faster detection of shared K-mer compared to all-against-all pair-wise sequence alignment executed with OLC algorithms [7]. However this approach leads to lower sensitivity and therefore leads to missing true overlaps [7]. We have found that the OLC methods (CAPRG, MIRA and PAVE) consistently outperformed VELVET regarding the total number of functional matches (BLAST) [25], number of unique matches and total number of unique matches against reference (Chicken) proteome for each Northern bobwhite (Figure 2) and Japanese quail (Figure 3) assemblies. Additionally, the reads that mapped to the reference genome either as single read or failed to assemble were reassembled (see Design and Implementation). This yielded an additional 1,156 and 1,271 putative coding regions for Northern bobwhite and Japanese quail, ranking CAPRG highest in gene-hunting for both species, as compared to other assemblers.

**Figure 4:**
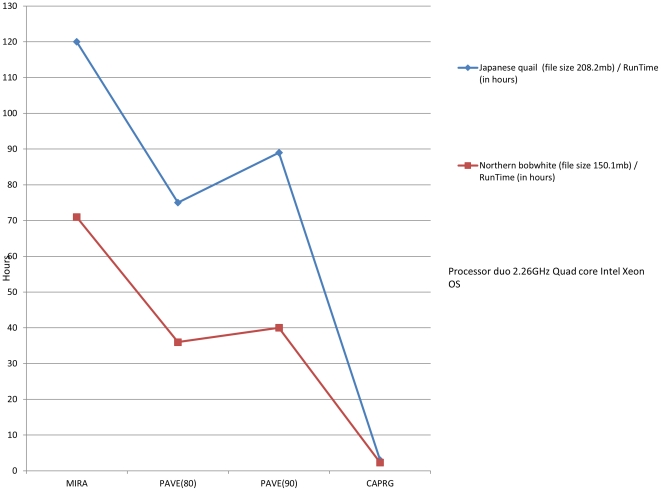
Runtime for each program. Assembly times represent execution on a computer with a duo 2.26 GHz Quad core Intel Xeon processor, 16 GB of RAM and 64 bit Snow Leopard v1.6 operating system.

In the presence of repeats, relaxed assembling parameters can result in false positive joins that could result in chimeric contigs [7]. One of the advantages of CAPRG is that it conducts fewer EST versus EST comparisons for alignment due to the limited window size strategy, as compared to the all-against-all, pair wise and K-mer approaches, thereby leading to a lower chance of producing chimeric contigs. This also leads to the reduction of contig inflation. The primary cause for contig inflation can be attributed to non-coding DNA sequenced from multiple haplotypes that are heterozygous due to lower selective constraints [3]. An illustration of contig inflation is observed where the highest number of contigs identified via assembly does not necessarily correspond with the identification of the highest number of unique protein-coding sequences (Figure 2 and Figure 3). Overall CAPRG produced a lower number of superfluous contigs and therefore completed assembling at fraction of runtime that was much faster than the other methods (Figure 4) with the exception of VELVET (finished in ∼20 mins). The trade off for the decreased computational time of VELVET is less robust sequence assembly when compared to the other assembly methods tested (Figure 2 and Figure 3). Given the restrictive window-based approach utilized in CAPRG, high quality assembly which rivals MIRA and PAVE can be achieved within a timeframe similar to VELVET using relatively modest computational power and memory overhead.

**Figure 5:**
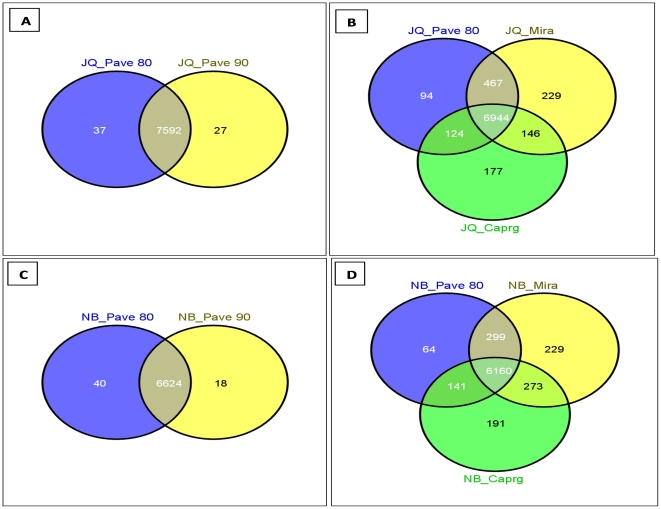
The extent of common protein sequences in different assemblies and parameters from Japanese quail datasets against chicken proteome database. Panel A represents the intra-assembling parameterization of PAVE at 80% and 90% identity and Panel B represents the inter-assembling comparison among PAVE, MIRA and CAPRG. Panel C represents the intra-assembling parameterization of PAVE at 80% and 90% identity and Panel D represents the inter-assembling comparison among PAVE, MIRA and CAPRG.

It has been observed that parameter space (parameterization) might be an important factor involved in the number of putative genes detected during sequence assembly [3]. However, given our datasets, we found that intra-assembling comparisons with parameter space did not lead to a higher number of diverse genes (Figure 5A). Most of the coding regions detected by a given assembler utilizing various parameters overlapped significantly. We compared the total number of genes identified by different assembly methods and found high numbers of putative genes that were identified uniquely within each assembly (Figure 5B). Similar trends were seen for the Northern bobwhite data (Figure 5C, 5D). The majority of assemblies were common (intersected) among these three assembly methods and a small relative percentage of genes were unique to each assembly method. Execution of all three assembly methods can therefore contribute to the identification of the maximum number of unique genes thereby increasing the ‘gene-hunting’ count (Figure 5B, 5D). Keeping these results in perspective, we suggest that multiple assemblies generated from intra-assembly parameterization might be useful. However, it would be more advantageous if two or more assembly methods are used for a transcriptomics study to generate a higher number of putative genes as discussed by Papanicolaou *et al.* (2009) [3] and Kumar *et al* (2010) [8].

One of the observations in transcriptome sequencing with NGS technologies is that full length transcripts are generally not sequenced though the transcripts are produced from whole mRNA [3]. This might be due to failure of an assembler to provide sufficient evidence of alignment due to high numbers of mismatches potentially due to limited conservation of non-coding regions, alternative splicing or multiple SNPs [3]. As a consequence, the ESTs may not overlap to assemble a contiguous sequence, giving rise to non-overlapping contigs, singletons or splits in gene [26], [27]. This issue of fragmentation not only leads to partial representation of a protein coding sequence but also redundancy in the assembled sequences where many of the contigs might actually represent the same protein (locus) leading to a redundancy factor that is introduced in assembly [3]. The redundancy index can be used to assess the quality of sequence assemblies. For example, low redundancy may reflect that the assembler is not sensitive to provide joins between reads leading to splits in assembly or that the assembler is able to recognize putative regions in same locus/identify SNPs that are not identified by other assemblers. If the average length of contigs for an assembly method are longer than those produced by an alternative assembly method, the assembler may be recognizing putative regions in the same locus rather than providing disjoined shorter sequence fragments which therefore results in higher redundancy. We calculated the redundancy index by dividing the total number of hits from the non-redundant (nr) protein database from NCBI by the total unique hits therefore providing the number of contigs that belong to the same locus for each organism. We found that the redundancy index of MIRA and CAPRG were highest followed by PAVE (Table S1). The average length of contigs in Japanese quail and in Northern bobwhite was highest for MIRA closely followed by CAPRG (Figure S1). Therefore, the performance of MIRA and CAPRG in detecting putative regions is likely maximized (Figure 2 and Figure 3) without sacrificing the sensitivity to provide the longest contig read lengths (Figure S1).

**Figure 6:**
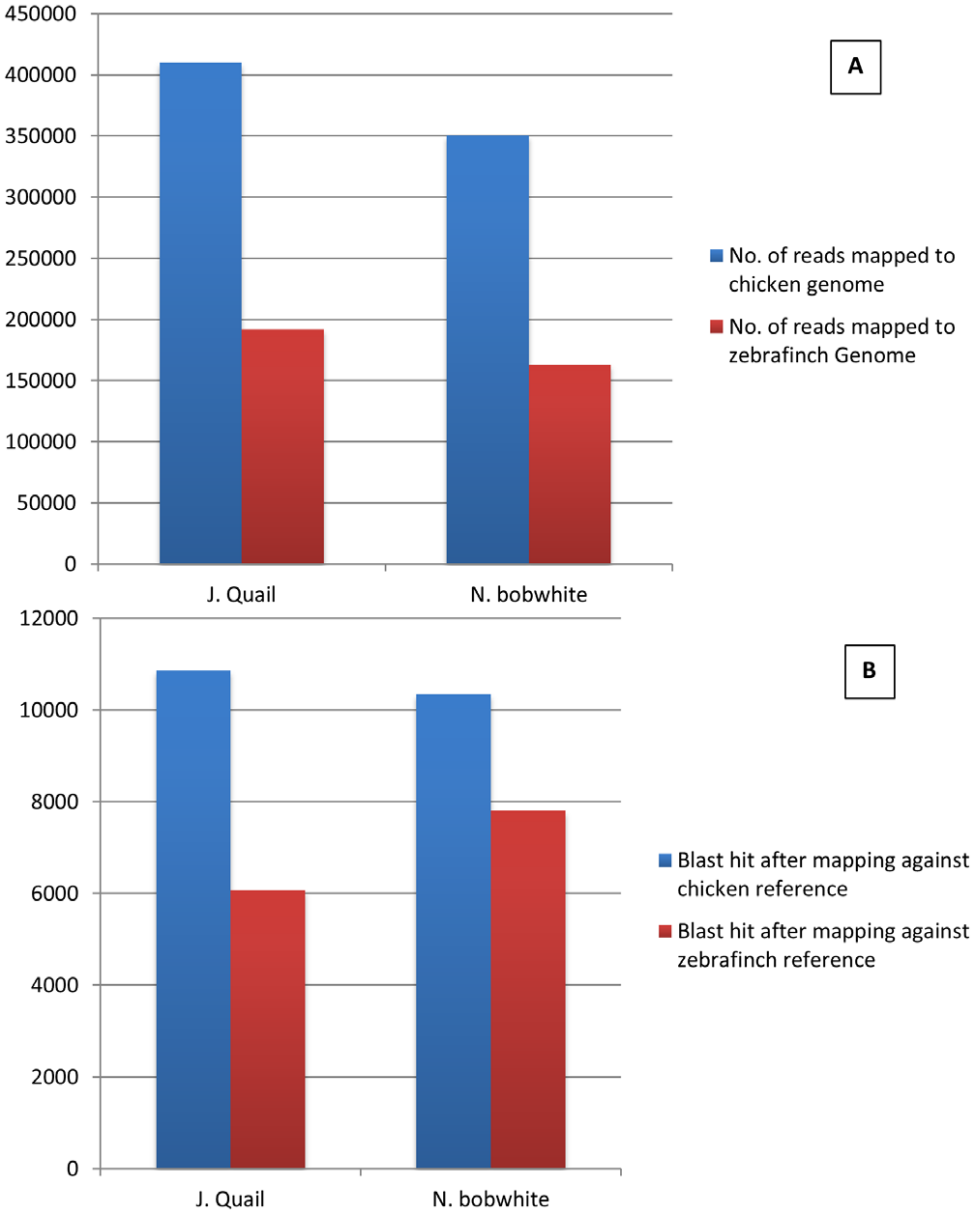
The effect of phylogenetic diversity on the assembling performance. Panel A Total number of reads mapped against chicken and zebra finch genome for *Coturnix japonica and Colinus virginianus*. Panel B Total number of unique hits against nr database for *Coturnix japonica and Colinus virginianus* mapped against chicken and zebra finch genome. *E-value* cutoff for all database searches was <10E-05. The abbreviation “nr” represents non-redundant protein database from NCBI.

Although CAPRG assembly presents many advantages to existing assembly methods, one caveat to this approach is the requirement of a reference genome that shares >94% identity with the genome of interest [28]. To evaluate the effect of phylogenetic diversity on the CAPRG assembling, we mapped the transcriptome reads against zebra finch (*Taeniopygia guttata*) genome [29], which belongs to a different taxonomic order (Passeriformes) compared to chicken, Japanese quail and Northern bobwhite (order Galliformes). We found that reads mapping to the zebra finch genome were reduced to nearly half when compared to the chicken reference genome for both Japanese quail and Northern bobwhite (Figure 6A). CAPRG generated a lower number of contigs from the decreased number of reads that mapped to zebra finch genome (21,212 for Northern bobwhite and 17,106 for zebra finch). The total number of unique hits against the nr database was reduced by 41% for Japanese quail and 25% for Northern bobwhite (Figure 6B). The selection of the phylogenetic neighbor effects the number of reads mapped to the reference genome thereby effecting contig generation and number of unique genes found. Based on this study, utilizing a close phylogenetic neighbor, preferably in the same family, generates optimal results.

**Figure 7:**
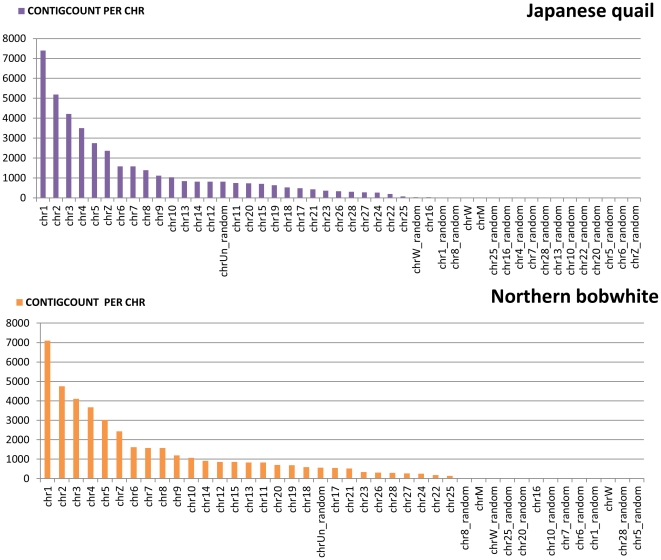
Distribution of contigs per chromosome for Japanese quail and Northern bobwhite against the chicken reference genome.

We examined the percentage of ESTs that are binned in each window in expectation to form contigs versus how many actually assembled to generate contigs. We found that a high percentage 95.4% (206157/216073) and 97% (249745/257566) of reads that were identified/binned for each window were successfully assembled as contigs (Table S2). Finally we visualized the distribution of all contigs that were binned across the expanse of chromosomes (Figure 7). We found that the distribution of both transcriptomes had a similar distribution against the chicken genome with chromosome 1, chromosome 2, chromosome 3, chromosome 4, and chromosome 5 representing the categories with highest number of contigs. This information can be utilized to further analyze contigs that are considered ‘unknown’ and are generally ignored in most studies thereby giving a broader picture of the entire transcriptome of a non-model organism.

## Materials and Methods

### Data

The transcriptomic datasets were generated for Northern bobwhite [30] and Japanese quail (Gust et al, Manuscript in preparation) by single-end Roche/454 GS-FLX sequencing. The Northern bobwhite is available at Short Read Archive (SRA) division [31] of GenBank under accession number SRA009460.5.

### Read Preprocessing

The ESTs were preprocessed by masking adaptors, base calling, and removal of unwanted sequences such as mitochondrial DNA, rDNA, homopolymers and other contaminants [32]. After preprocessing, the datasets for Northern bobwhite and Japanese quail were assembled using CAPRG and the pre-existing assembly tools PAVE (version 1_0), MIRA (version 2.9) and VELVET (version 0.7.56).

### Computing Infrastructure

The assemblies were performed using a computer with duo 2.26 GHz Quad core Intel Xeon processors (Intel Corporation, Santa Clara, CA) and 16 GB RAM with the 64 bit Snow Leopard v1.6 (Apple Computer Inc. Cupertino, CA) operating system. The CAPRG pipeline was implemented with PERL 5.10.0, and BioPERL 1.6 script programs interfacing with MySQL 5.4.3 database (www.mysql.com) through PERL-DBI. Other dependencies include Burrows-Wheeler Alignment (BWA 0.5.7) [15], SAMTools [33] and CAP3 [4]. The Chicken proteome was downloaded in Aug'2010 from Entrez in fasta format [34]. The chicken reference genome build May 2006 and Zebra Finch genome build 2008 was downloaded from Golden Path [35].

The default settings for assembly tools were utilized for MIRA, PAVE and VELVET (using the “long-read switch” appropriate for GS-FLX data) except as mentioned in the Results and Discussion section. The two-step process of alignment and assembling in CAPRG uses BWA-SW default settings and assembling with CAP3 at 90% identity and overlap of 20 bp. Single reads might align to multiple chromosomal positions with different mapping quality (MAPQ) values. Only the read with maximum mapping quality representing a unique locus was imported into the MySQL database.

The assembling process begins with ordering the reads according to its unique position on each chromosome iteratively. All reads with overlapping junction based on chromosomal position with the previous read are binned in a single window and assembled. A new bin is created each time the chromosomal position of the next read falls beyond the window (the sum total of previous read's chromosomal position and the read length). The assembling is performed with parameters as discussed above, and can be considered optimal for the reduced read population size in each window (as against all-against-all population size). The identity parameter (90%) is kept stringent as we expect the reads binned in a window to be highly identical and the overlap length is kept low, the minimum allowed by CAP3, to allow maximum joins among reads to form longer contigs. The parameter space is not applied as 95.4% and 97% of Japanese quail and Northern bobwhite binned reads were successfully assembled and will not affect the overall results. Additionally, all reads that mapped to reference genome either singly or failed to assemble and resulted in singlets (45,291 and 88,000 for Northern bobwhite and Japanese quail respectively) were assembled with CAP3 at 90% identity and 40 bp overlap.

Investigation of final results for the finished assembly generated for each assembly method included the exclusion of all singlets and all contigs with read lengths >200 bp were used for comparison of different methods. Resultant contigs for each assembly method were annotated against the non-redundant (nr) protein database from NCBI using Parallel Blast [36] with high performance computing (HPC) (albacore.st.usm.edu) and BLAST [25] programs against chicken database.

### Availability

The project is available at http://code.google.com/p/caprg/. We plan to implement the multithreaded version of CAPRG that will further reduce the computational cost. This will especially help to assemble Illumina reads (>150) that have high depth and read number (∼10 M).

## Supporting Information

Figure S1 Comparison of average length of contigs of different assemblies.
(DOC)

Table S1 Redundancy index of various sequence assembly methods.
(XLS)

Table S2 Breakdown of ESTs binned per window and actual ESTs assembled per window.
(XLS)
