# Supplementary figures and images for: CAPRG: Sequence Assembling Pipeline for Next Generation Sequencing of Non-Model Organisms
Source: PLoS One. 2012 Feb 3;7(2):e30370. doi: 10.1371/journal.pone.0030370 (PMC3272009; doi:10.1371/journal.pone.0030370)

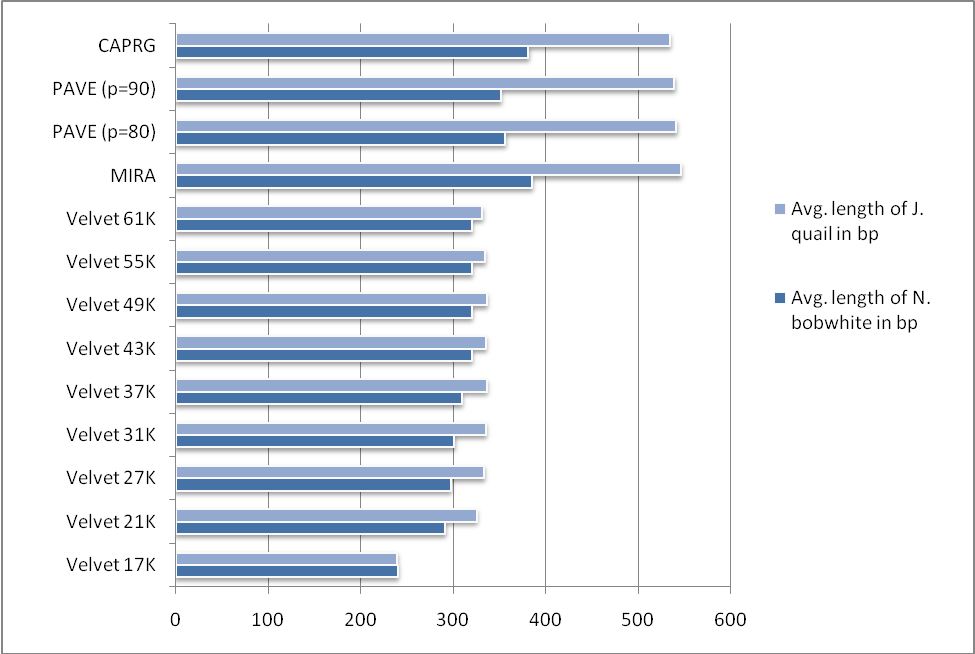


Suppl Fig 1. Comparison of average length of contigs of different assemblies

Supplement: Figure S1 — Comparison of average length of contigs of different assemblies. (DOC) [file pone.0030370.s001.doc]
